# {[(*E*)-(1,3-Benzodioxol-5-yl)methyl­idene]amino}thio­urea

**DOI:** 10.1107/S2056989024000033

**Published:** 2024-01-09

**Authors:** Ernesto Mesto, Coco K. Y. A. Okio, Maria Alejandra Lemus, Emanuela Schingaro

**Affiliations:** a Dipartimento di Scienze della Terra e Geoambientali., Università degli Studi di Bari Aldo Moro, Via E. Orabona 4, 701125 Bari, Italy; b Departamento de Quimica, Facultad de Ciencias, Universidad Nacional de Colombia, Carerra 30 No 45-03, Bogotá, Colombia; Institute of Chemistry, Chinese Academy of Sciences

**Keywords:** crystal structure, thio-semicarbazone, three-dimensional network

## Abstract

The synthesis and crystallographic analysis of the title compound, C_9_H_9_N_3_O_2_S, are reported. The compound crystallizes in the monoclinic space group *P*2_1_/*c*, revealing characteristic bond lengths and angles typical of thio­semicarbazone groups. The supra­molecular organization primarily arises from hydrogen bonding and π–π stacking inter­actions, leading to distinctive dimeric formations.

## Abstract 2

The synthesis and crystallographic analysis of [(*E*)-1,3-benzodioxol-5-yl­methyl­idene­amino]­thio­urea is reported, offering a comprehensive exploration of its structural features and supra­mol­ecular arrangements within the crystal.

## 1. Chemical context

The group of thio­semicarbazone Schiff bases, capable of coordinating with metal centers through nitro­gen and sulfur atoms, has garnered significant recent attention (Cortés *et al.*, 2011 ▸; Singh *et al.*, 2016 ▸) due to the exhibited biological and pharmacological properties, such as anti­bacterial and anti­viral activities (Hu *et al.*, 2006 ▸). This study focuses on describing the synthesis and the analysis of the crystal structure of the title mol­ecule. 
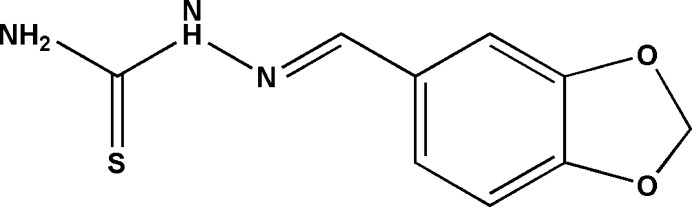


## 2. Structural commentary

**Figure 1:**
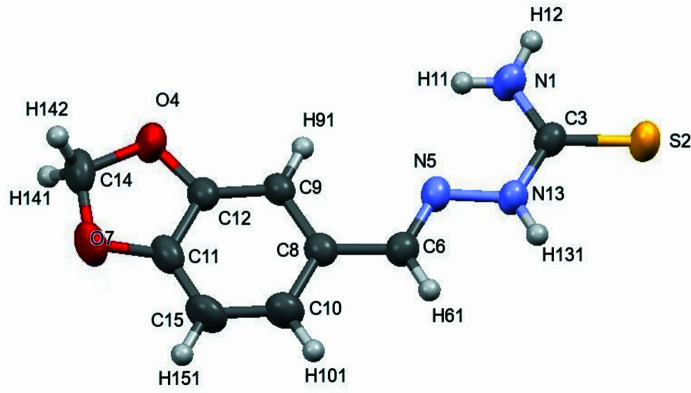
Labelling scheme and structure of **I**. Displacement ellipsoids are drawn at the 50% probability level.

The target compound (**I**) crystallizes in the monoclinic space group *P*2_1_/*c* with one mol­ecule in the asymmetric unit (*Z* = 4). A view of the mol­ecule is shown in Fig. 1 ▸. Selected bond lengths and angles for the product are listed in Table 1 ▸. All bond lengths exhibit typical values (Dias *et al.*, 2017 ▸).

## 3. Supra­molecular features

**Figure 2:**
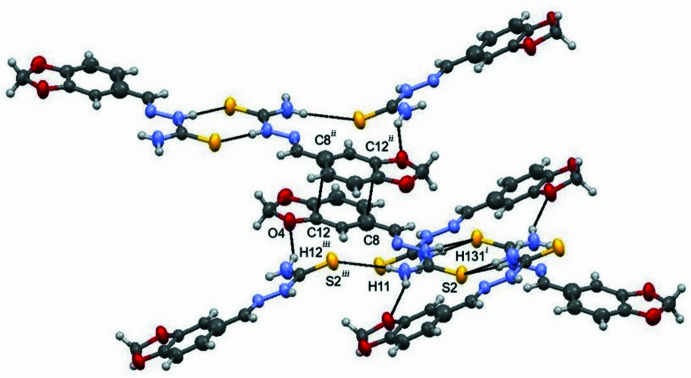
The mol­ecular dimers of mol­ecule **I** (arbitrary view). Hydrogen bonds and π–π stacking inter­actions are indicated by dotted lines. [Symmetry codes: (i) 1 − *x*, −*y*, 1 − *z*; (ii) 2 − *x*, −*y*, 1 − *z*; (iii) 1 − *x*, 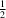 + *y*, 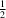 − *z*.]

The supra­molecular arrangements of **I** primarily result from classical and non-classical hydrogen bonds and π–π stacking inter­actions. These contacts were recognized by *Mercury 2022* (Macrae *et al.*, 2020 ▸; sum of van der Waals radii plus 0.1 Å). The hydrogen-bonding geometry is listed in Table 2 ▸, and the packing of mol­ecules viewed down along the *c* axis is shown in Fig. 2 ▸. Together, the hydrogen-bonding inter­actions lead to the formation of a two-dimensional network parallel to (100). The mol­ecules at (*x*, *y*, *z*) and (2 − *x*, −*y*, 1 − *z*) are components of dimers centered at (1, 0, ½), while the separation between the aryl ring centroids is 3.778 (2) Å, indicating π–π stacking interactions between the aromatic ring systems.

## 4. Database Survey

Six crystal structures, authored by different researchers and featuring the [(*E*)-1,3-benzodioxol-5-yl­meth­ylidene­amino]­thio­urea fragment (piperonal, thio­semicarbazone), have been documented in the Cambridge Structural Database (CSD, WebCSD search December 2023; Groom *et al.*, 2016 ▸). The crystal structure here under discussion can be considered the parent compound among those reported in the CSD. These other structures show substitutions in the imine hydrogen with methyl (de Oliveira *et al.*, 2013 ▸, 2015*a* ▸,*b* ▸) or nitro group (Dias *et al.*, 2017 ▸), and in the amidic hydrogen with methyl (de Oliveira *et al.*, 2015*b* ▸), ethyl (Dias *et al.* 2017 ▸; de Oliveira *et al.*, 2015*a* ▸), and phenyl (Dias *et al.* 2017 ▸) radicals. Some of these structures crystallize in the triclinic space group *P* 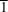 , while others in the monoclinic space group *P*2_1_/*c.* Finally, Beckford *et al.* (2011 ▸), provide detailed information on the crystal structure of [(η^6^-p-cymene)Ru(*p*PhTSC)Cl]Cl, which crystallizes in the monoclinic space group *P*2_1_/*n*. All of the structures reveal co-planar arrangements of the piperonal thio­semicarbazone portion along with π–π and hydrogen-bonding inter­actions.

## 5. Synthesis and crystallization

The synthesis of the Schiff base ligand (1,3-benzodioxol-5-ylformaldehyde) thio­semicarbazone was performed according to a previously published procedure (Casas *et al.*, 2015 ▸). Piperonal (1.00 g, 6.66 mmol) and thio­semicarbazide (0.61 g, 6.66 mmol) were dissolved in ethanol and stirred under reflux for 2 h. Upon cooling, the solvent was removed and the remaining solid was recrystallized from ethanol/di­chloro­methane. Yellowish crystals suitable for X-ray diffraction were grown by slow evaporation after a couple of weeks, yield 78%; m.p. 409–411) . FT–IR (ATR, cm^−1^): 3323 ν(N—H), 1585 ν(C=N), 1090 ν(N—N), 931 ν(C=S).

## 6. Refinement details

Crystal data, data collection and structure refinement details are summarized in Table 3 ▸. The H atoms were all located in difference maps, but those attached to carbon atoms were repositioned geometrically. The H atoms were initially refined with soft restraints on the bond lengths and angles to regularize their geometry (C—H in the range 0.93–0.98 Å, N—H = 0.86 Å, O—H = 0.82 Å) and *U*_iso_(H) (in the range 1.2–1.5 times *U*_eq_ of the parent atom), after which the positions were refined with riding constraints.

## Supplementary Material

Crystal structure: contains datablock(s) global, I. DOI: 10.1107/S2056989024000033/nx2003sup1.cif

Structure factors: contains datablock(s) I. DOI: 10.1107/S2056989024000033/nx2003Isup2.hkl

Supporting information file. DOI: 10.1107/S2056989024000033/nx2003Isup3.mol

CCDC reference: 2190589

Additional supporting information:  crystallographic information; 3D view; checkCIF report

## Figures and Tables

**Table 1:** Selected geometric parameters (Å, °)

N1—C3	1.3281 (17)	N5—C6	1.2793 (17)
S2—C3	1.6899 (13)	N5—N13	1.3800 (15)
C3—N13	1.3414 (17)		
			
S2—C3—N1	122.87 (11)	N1—C3—N13	116.82 (12)
S2—C3—N13	120.31 (10)	C6—N5—N13	117.07 (11)

**Table 2:** Hydrogen-bond geometry (Å, °)

*D*—H⋯*A*	*D*—H	H⋯*A*	*D*⋯*A*	*D*—H⋯*A*
N1—H11⋯N5	0.85 (2)	2.23 (2)	2.6034 (17)	107 (1)
N1—H11⋯S2^i^	0.85 (2)	2.84 (2)	3.4805 (14)	134 (1)
N1—H12⋯O4^ii^	0.86 (1)	2.32 (2)	3.112 (2)	153 (2)
N13—H131⋯S2^iii^	0.86 (1)	2.50 (1)	3.3550 (12)	172 (1)

Symmetry codes: (i) 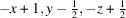 ; (ii) 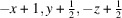 ; (iii) 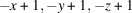 .

**Table 3:** Experimental details

Crystal data	
Chemical formula	C_9_H_9_N_3_O_2_S
*M*_r_	223.26
Crystal system, space group	Monoclinic, *P*2_1_/*c*
Temperature (K)	293
*a*, *b*, *c* (Å)	7.1189 (2), 10.9687 (2), 13.0678 (3)
β (°)	100.426 (2)
*V* (Å^3^)	1003.55 (4)
*Z*	4
Radiation type	Mo *K*α
μ (mm^−1^)	0.31
Crystal size (mm)	0.58 × 0.34 × 0.17

Data collection	
Diffractometer	Bruker APEXII
Absorption correction	Multi-scan (*SADABS*; Krause *et al.*, 2015 ▸)
*T*_min_, *T*_max_	0.88, 0.95
No. of measured, independent and observed [*I* > 2.0σ(*I*)] reflections	19600, 3818, 2447
*R*_int_	0.000
(sin θ/λ)_max_ (Å^−1^)	0.770

Refinement	
*R*[*F* ^2^ > 2σ(*F* ^2^)], *wR*(*F* ^2^), *S*	0.040, 0.104, 0.95
No. of reflections	3810
No. of parameters	148
No. of restraints	10
H-atom treatment	H atoms treated by a mixture of independent and constrained refinement
Δρ_max_, Δρ_min_ (e Å^−3^)	0.38, −0.32

Computer programs: *APEX2* and *SAINT* (Bruker, 2006 ▸), *SUPERFLIP* (Palatinus & Chapuis, 2007 ▸), *CRYSTALS* (Betteridge *et al.*, 2003 ▸) and *Mercury* (Macrae *et al.*, 2020 ▸).

## supplementary crystallographic information

### Crystal data

**Table:**

C_9_H_9_N_3_O_2_S	*F*(000) = 464
*M**_r_* = 223.26	*D*_x_ = 1.478 Mg m^−^^3^
Monoclinic, *P*2_1_/*c*	Mo *K*α radiation, λ = 0.71073 Å
Hall symbol: -P 2ybc	Cell parameters from 3399 reflections
*a* = 7.1189 (2) Å	θ = 3.2–27.6°
*b* = 10.9687 (2) Å	µ = 0.31 mm^−^^1^
*c* = 13.0678 (3) Å	*T* = 293 K
β = 100.426 (2)°	Tabular, colourless
*V* = 1003.55 (4) Å^3^	0.58 × 0.34 × 0.17 mm
*Z* = 4	

### Data collection

**Table:**

Bruker APEXII diffractometer	2447 reflections with *I* > 2.0σ(*I*)
Graphite monochromator	*R*_int_ = 0.000
φ & ω scans	θ_max_ = 33.2°, θ_min_ = 3.2°
Absorption correction: multi-scan (SADABS; Krause *et al.*, 2015)	*h* = −10→10
*T*_min_ = 0.88, *T*_max_ = 0.95	*k* = −16→16
19600 measured reflections	*l* = −20→20
3818 independent reflections	

### Refinement

**Table:**

Refinement on *F*^2^	Primary atom site location: other
Least-squares matrix: full	Hydrogen site location: difference Fourier map
*R*[*F*^2^ > 2σ(*F*^2^)] = 0.040	H atoms treated by a mixture of independent and constrained refinement
*wR*(*F*^2^) = 0.104	Method = Modified Sheldrick *w* = 1/[σ^2^(*F*^2^) + ( 0.04*P*)^2^ + 0.31*P*] , where *P* = (max(*F*_o_^2^,0) + 2*F*_c_^2^)/3
*S* = 0.95	(Δ/σ)_max_ = 0.001
3810 reflections	Δρ_max_ = 0.38 e Å^−^^3^
148 parameters	Δρ_min_ = −0.32 e Å^−^^3^
10 restraints	

### Fractional atomic coordinates and isotropic or equivalent isotropic displacement parameters (Å^2^)

**Table:**

	*x*	*y*	*z*	*U*_iso_*/*U*_eq_	
N1	0.4666 (2)	0.28730 (12)	0.27357 (10)	0.0521	
S2	0.41135 (7)	0.51407 (3)	0.33300 (3)	0.0508	
C3	0.4856 (2)	0.36798 (11)	0.35054 (10)	0.0378	
O4	0.82417 (18)	−0.24246 (9)	0.43350 (9)	0.0596	
N5	0.62732 (16)	0.20875 (9)	0.45653 (9)	0.0383	
C6	0.70095 (19)	0.17336 (12)	0.54832 (10)	0.0392	
O7	0.96272 (18)	−0.30444 (10)	0.59886 (10)	0.0619	
C8	0.76809 (19)	0.04823 (12)	0.56576 (10)	0.0370	
C9	0.75307 (19)	−0.03330 (11)	0.48105 (10)	0.0375	
C10	0.8517 (2)	0.01062 (15)	0.66540 (11)	0.0497	
C11	0.9050 (2)	−0.18475 (13)	0.60146 (12)	0.0451	
C12	0.82203 (19)	−0.14781 (12)	0.50257 (11)	0.0389	
N13	0.56921 (18)	0.32877 (10)	0.44507 (9)	0.0405	
C14	0.9020 (3)	−0.34471 (13)	0.49501 (14)	0.0556	
C15	0.9220 (3)	−0.10782 (16)	0.68462 (12)	0.0560	
H61	0.7130	0.2267	0.6055	0.0484*	
H91	0.6977	−0.0091	0.4133	0.0462*	
H101	0.8592	0.0663	0.7228	0.0604*	
H141	1.0143	−0.3736	0.4670	0.0677*	
H142	0.8011	−0.4081	0.4918	0.0665*	
H151	0.9779	−0.1327	0.7520	0.0688*	
H12	0.395 (3)	0.3055 (15)	0.2155 (12)	0.0633 (19)*	
H131	0.577 (2)	0.3758 (12)	0.4987 (10)	0.0488 (18)*	
H11	0.498 (3)	0.2137 (13)	0.2878 (12)	0.0632 (19)*	

### Atomic displacement parameters (Å^2^)

**Table:**

	*U*^11^	*U*^22^	*U*^33^	*U*^12^	*U*^13^	*U*^23^
N1	0.0787 (10)	0.0371 (6)	0.0379 (6)	0.0057 (6)	0.0032 (6)	−0.0042 (5)
S2	0.0777 (3)	0.03268 (16)	0.04106 (19)	0.00766 (16)	0.00842 (17)	0.00264 (13)
C3	0.0442 (7)	0.0321 (6)	0.0381 (6)	−0.0018 (5)	0.0103 (5)	−0.0016 (5)
O4	0.0838 (9)	0.0308 (5)	0.0576 (7)	0.0074 (5)	−0.0046 (6)	0.0013 (4)
N5	0.0428 (6)	0.0299 (5)	0.0426 (6)	0.0028 (4)	0.0086 (5)	−0.0001 (4)
C6	0.0410 (7)	0.0381 (6)	0.0392 (7)	0.0004 (5)	0.0090 (5)	−0.0018 (5)
O7	0.0703 (8)	0.0422 (6)	0.0685 (8)	0.0094 (5)	−0.0002 (6)	0.0207 (5)
C8	0.0353 (6)	0.0380 (6)	0.0376 (6)	−0.0017 (5)	0.0067 (5)	0.0040 (5)
C9	0.0385 (7)	0.0346 (6)	0.0374 (6)	−0.0010 (5)	0.0009 (5)	0.0055 (5)
C10	0.0577 (9)	0.0547 (8)	0.0353 (7)	0.0021 (7)	0.0048 (6)	0.0027 (6)
C11	0.0417 (7)	0.0405 (7)	0.0517 (8)	0.0005 (6)	0.0045 (6)	0.0161 (6)
C12	0.0386 (7)	0.0334 (6)	0.0430 (7)	−0.0029 (5)	0.0027 (5)	0.0046 (5)
N13	0.0534 (7)	0.0310 (5)	0.0362 (6)	0.0055 (5)	0.0060 (5)	−0.0031 (4)
C14	0.0565 (9)	0.0339 (7)	0.0760 (11)	0.0052 (6)	0.0107 (8)	0.0129 (7)
C15	0.0631 (10)	0.0617 (10)	0.0390 (7)	0.0037 (8)	−0.0016 (7)	0.0164 (7)

### Geometric parameters (Å, º)

**Table:**

N1—C3	1.3281 (17)	C8—C9	1.4120 (18)
N1—H12	0.858 (14)	C8—C10	1.3926 (19)
N1—H11	0.849 (14)	C9—C12	1.3592 (17)
S2—C3	1.6899 (13)	C9—H91	0.941
C3—N13	1.3414 (17)	C10—C15	1.399 (2)
O4—C12	1.3776 (17)	C10—H101	0.961
O4—C14	1.4318 (17)	C11—C12	1.3809 (19)
N5—C6	1.2793 (17)	C11—C15	1.364 (2)
N5—N13	1.3800 (15)	N13—H131	0.864 (13)
C6—C8	1.4576 (18)	C14—H141	0.990
C6—H61	0.941	C14—H142	0.995
O7—C11	1.3779 (18)	C15—H151	0.939
O7—C14	1.418 (2)		
			
C3—N1—H12	118.5 (11)	C15—C10—H101	118.8
C3—N1—H11	118.8 (11)	O7—C11—C12	109.60 (14)
H12—N1—H11	120.3 (15)	O7—C11—C15	128.71 (13)
S2—C3—N1	122.87 (11)	C12—C11—C15	121.69 (13)
S2—C3—N13	120.31 (10)	C11—C12—O4	109.78 (12)
N1—C3—N13	116.82 (12)	C11—C12—C9	122.83 (13)
C12—O4—C14	105.76 (11)	O4—C12—C9	127.39 (12)
C6—N5—N13	117.07 (11)	N5—N13—C3	118.70 (11)
N5—C6—C8	119.96 (12)	N5—N13—H131	120.5 (9)
N5—C6—H61	121.3	C3—N13—H131	120.5 (9)
C8—C6—H61	118.7	O4—C14—O7	108.25 (12)
C11—O7—C14	106.25 (11)	O4—C14—H141	107.4
C6—C8—C9	119.84 (12)	O7—C14—H141	109.1
C6—C8—C10	119.85 (13)	O4—C14—H142	108.7
C9—C8—C10	120.28 (13)	O7—C14—H142	110.5
C8—C9—C12	116.73 (12)	H141—C14—H142	112.7
C8—C9—H91	121.3	C10—C15—C11	117.03 (13)
C12—C9—H91	122.0	C10—C15—H151	121.0
C8—C10—C15	121.44 (14)	C11—C15—H151	121.9
C8—C10—H101	119.7		

### Hydrogen-bond geometry (Å, º)

**Table:**

*D*—H···*A*	*D*—H	H···*A*	*D*···*A*	*D*—H···*A*
N1—H11···N5	0.85 (2)	2.23 (2)	2.6034 (17)	107 (1)
N1—H11···S2^i^	0.85 (2)	2.84 (2)	3.4805 (14)	134 (1)
N1—H12···O4^ii^	0.86 (1)	2.32 (2)	3.112 (2)	153 (2)
N13—H131···S2^iii^	0.86 (1)	2.50 (1)	3.3550 (12)	172 (1)

Symmetry codes: (i) −*x*+1, *y*−1/2, −*z*+1/2; (ii) −*x*+1, *y*+1/2, −*z*+1/2; (iii) −*x*+1, −*y*+1, −*z*+1.
